# A Case of Follicular Dendritic Cell Sarcoma of the Palatine Tonsil That Developed as a Radiation-Induced Sarcoma

**DOI:** 10.1155/crot/8861715

**Published:** 2025-10-19

**Authors:** Alyssa Yoshida, Takuya Mikoshiba, Mariko Sekimizu, Shintaro Nakamura, Ryoto Nagai, Miho Kawaida, Katsura Emoto, Hiroyuki Ozawa

**Affiliations:** ^1^Department of Otolaryngology, Head and Neck Surgery, Keio University School of Medicine, 35 Shinanomachi Shinjuku-ku, Tokyo 160-8582, Japan; ^2^Division of Diagnostic Pathology, Keio University Hospital, 35 Shinanomachi Shinjuku-ku, Tokyo 160-8582, Japan

**Keywords:** follicular dendritic cell sarcoma, radiation-induced sarcoma, tonsil tumor

## Abstract

Follicular dendritic cell sarcoma is a rare malignant tumor originating from follicular dendritic cells. We present the first report of such a sarcoma of the palatine tonsils that developed as a radiation-induced sarcoma. A 78-year-old man, who had undergone chemoradiotherapy for hypopharyngeal and esophageal cancer 7 years prior, presented with discomfort during swallowing. Endoscopic pharyngeal examination revealed a tumor in the right palatine tonsil which was histopathologically diagnosed as a follicular dendritic cell sarcoma. The tumor also met the diagnostic criteria for radiation-induced sarcoma. Positron emission tomography/computed tomography revealed abnormal uptake in the right palatine tonsil and bilateral cervical lymph nodes; no distant metastases were detected. The patient underwent tumor resection using the mandibular swing approach, pharyngeal reconstruction using an anterolateral thigh flap, bilateral neck lymph node dissection, and tracheostomy. Postoperative radiotherapy was not administered because of previous irradiation; however, no apparent recurrence or metastasis was observed 4 years after surgery. Follicular dendritic cell sarcoma can develop over an extended period after previous irradiation. Extended resection with a negative margin may be pivotal in treating follicular dendritic cell sarcoma when postoperative radiotherapy cannot be administered.

## 1. Introduction

Follicular dendritic cell sarcoma (FDCS) is a rare neoplasm, first reported by Monda et al., that originates from follicular dendritic cells and antigen-presenting cells within lymph follicles [1, 2]. FDCS occurs across various age groups and primarily affects young to middle-aged adults of both sexes [2]. Although FDCS predominantly occurs within the lymph nodes, it can occasionally occur at extranodal sites [3]. Histological examination is essential for its diagnosis, and positivity for dendritic cell markers such as CD21, CD23, and CD35 on immunological staining is helpful [2, 4]. The standard treatment for FDCS remains controversial because of its rarity; however, most reports tend to recommend radical surgery [3, 5, 6], with some suggesting postoperative radiotherapy (PORT) [5, 7].

The carcinogenic potential of radiation has long been acknowledged, and Beck et al. first reported radiation-induced sarcoma (RIS) in bones [8]. The criteria for RIS were first reported by Cahan et al. and, since then, several revisions to the criteria have been proposed [8–12]. Generally, the requirements include the following: (1) sarcoma originating within the irradiated field, (2) sarcoma must have been histologically confirmed and present a different histological type from the primary tumor, and (3) a latency of several years between radiation exposure and subsequent diagnosis of the sarcoma [8–12]. Head and neck lesions have been reported in patients with RIS [10, 13]; however, few reports have described an FDCS developing as an RIS [13]. Herein, we present the first report of an FDCS of the palatine tonsil that developed as an RIS.

## 2. Case Presentation

**Figure 1:**
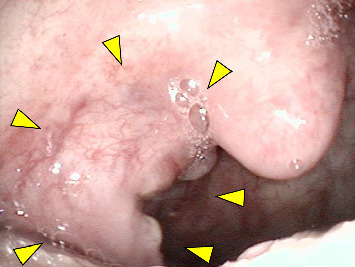
Endoscopic examination of the pharynx reveals a tumor with ulceration in the right palatine tonsil (arrowhead).

**Figure 2:**
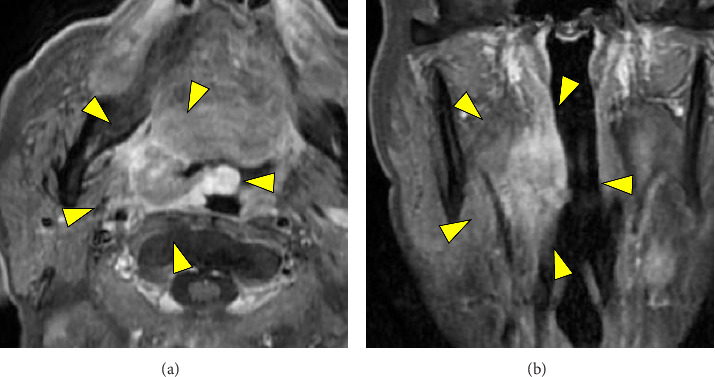
Contrast-enhanced magnetic resonance imaging reveals a mass in the right palatine tonsil (arrowhead). The tumor shows marginal enhancement and invades the surrounding pharyngeal constrictor muscle ((a) axial view; (b) coronal view).

**Figure 3:**
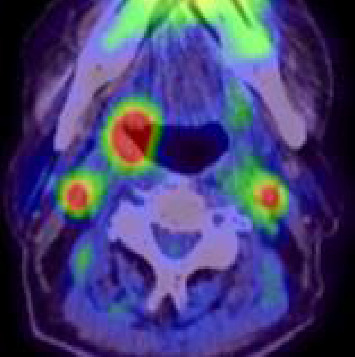
Positron emission tomography/computed tomography shows abnormal uptake in the right palatine tonsil and bilateral cervical lymph nodes.

A 78-year-old man presented with a chief complaint of discomfort during swallowing. The patient had undergone chemoradiotherapy for squamous cell carcinoma of the hypopharynx and esophagus 7 years prior, with no signs of recurrence or metastasis. Endoscopic pharyngeal examination revealed an ulcerative mass in the right palatine tonsil (Figure 1). Contrast-enhanced magnetic resonance imaging revealed a mass with marginal enhancement in the right palatine tonsil and invasion of the surrounding pharyngeal constrictor muscle (Figures 2(a) and 2(b)). Positron emission tomography/computed tomography revealed abnormal uptake in the right palatine tonsil and bilateral cervical lymph nodes; no distant metastases were detected (Figure 3). Despite performing biopsies twice for the palatine tonsil tumor, a definitive diagnosis remained challenging. FDCS was considered in the differential diagnosis; however, the diagnosis could not be confirmed because the tumor cells were only partially positive for CD21 and negative for CD23 and CD35. Because the tumor cells were positive for LCA and CD4, T-cell lymphoma was considered in the differential diagnosis; however, the other T-cell markers, including CD3 and CD7, were negative. In addition, weak positivity for AE1/AE3 and CAM5.2, as well as positivity for Claudin 4 did not rule out the possibility of carcinoma. Therefore, the patient underwent debulking surgery under general anesthesia to obtain adequate tissue for a definitive diagnosis.

**Figure 4:**
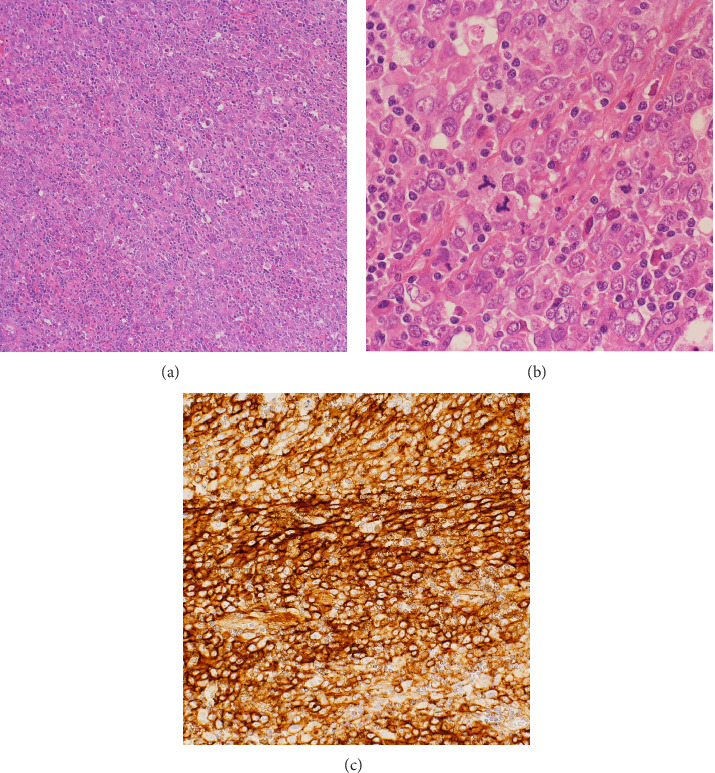
Hematoxylin-eosin staining reveals the proliferation of large, atypical cells with enlarged nuclei and small lymphocytes distributed in the background ((a) low-power view; (b) high-power view). Immunohistochemistry shows that the tumor cells were positive for CD21 (c).

Histopathological examination of the debulking surgery specimens revealed the proliferation of large, atypical cells with enlarged nuclei and small lymphocytes distributed in the background on hematoxylin-eosin staining. The boundaries of the tumor cells were unclear (Figures 4(a) and 4(b)). The tumor cells were diffusely positive for CD21, a follicular dendritic cell marker (Figure 4(c)), but negative for CD23 and CD35. Similar to the initial biopsy, the tumor cells were positive for LCA and CD4; however, they were negative for the other T cell markers, including CD3 and CD7, ruling out the diagnosis of T-cell lymphoma. Only a limited number of epithelial markers, including CK AE1/AE3, showed staining. The tumor cells were negative for CD5, CD8, CD10, CD20, CD23, CD79a, CD56, CD68, CD1a, and 34βE12. Ki-67 had an expression rate of more than 90%. According to the immunochemical findings, the patient was diagnosed with FDCS. Additionally, based on previous reports, the tumor was histopathologically diagnosed as FDCS according to the RIS criteria [8–12].

The patient underwent radical resection of the tumor with a wide safety margin using the mandibular swing approach, bilateral neck lymph node dissection, and tracheostomy. The incision was made anterior to the palatopharyngeal arch, retromolar area, lateral oropharyngeal wall, and upper edge of the soft palate. A third of the base of the tongue was included in the resected specimen. The internal carotid artery was followed to the upper edge of the oropharynx from the neck and the tumor was resected en bloc. The defect was reconstructed with an anterolateral thigh flap. After surgery, the patient experienced dysphagia; however, swallowing rehabilitation through effortful swallowing exercises led to improvement. The patient was discharged 51 days after surgery with no other complications. Postoperative pathology revealed negative margins for the primary tumor of the right palatine tonsil and positive findings of bilateral cervical lymph node metastasis. The patient could not undergo adjuvant radiotherapy because of previous irradiation. No additional treatment was administered and, 4 years after surgery, no apparent recurrence or metastasis was observed.

## 3. Discussion

FDCS is a rare tumor, with at least 137 cases reported in the head and neck region and approximately 30 in the palatine tonsils [3, 6, 13]. In particular, FDCS developing as RIS is extremely rare, with only one case reported in the parotid gland [13]. To the best of our knowledge, this is the first case of FDCS of the palatine tonsil that developed as an RIS. Acknowledging that FDCS can develop after head and neck irradiation is crucial.

Immunostaining for follicular dendritic cell markers is important for diagnosing FDCS, which is often histologically confused with mesenchymal and lymphoid neoplasms [14]. In our case, the patient underwent an outpatient biopsy; however, a diagnosis could not be made because the tumor cells were only partially positive for CD21 and negative for other dendritic cell markers such as CD23 and CD35 [15]. Debulking surgery under general anesthesia was performed to obtain adequate tissue for examination, which revealed that the tumor cells were diffusely positive for CD21, leading to the diagnosis of FDCS. Immunohistochemical evaluation of adequate tissue samples was warranted to confirm this diagnosis. The tumor cells were positive for CD21 but, in the present case, negative for CD23 and CD35. In previous cases of FDCS, not all follicular dendritic cell markers were consistently positive: some cases exhibited partial positivity [16]. Consistent with that report, FDCS was diagnosed in our case based on CD21 positivity [16].

RIS can develop over a wide range of periods after irradiation. In a previous report of 67 patients with radiation-induced soft tissue sarcomas, the period over which RIS developed after radiotherapy ranged from 3 to 36 years (median, 11 years) [17]. Regarding head and neck lesions, radiation-induced FDCS can develop in the parotid gland 20 years after irradiation [13]. In our case, the period between the previous irradiation and the development of FDCS was approximately 7 years, which is consistent with other histological types. Given that RIS can develop over a long-term course after radiation therapy, regular follow-ups are warranted to evaluate its development.

A standard therapy for FDCS of the head and neck has not yet been established. However, Pang et al. reported that among patients with FDCS arising in the head and neck, those who underwent surgery and PORT had significantly lower local recurrence rates than those who underwent surgery alone [7]. Chera et al. suggested that extended resection plus PORT should be performed for extranodal tumors [5]. In the present case, PORT could not be administered because the patient had a history of radiotherapy. However, the patient did not experience recurrence or metastasis, implying that extended resection with a negative margin could be sufficient to control tumors without PORT. On the other hand, evidence regarding the efficacy of chemotherapy for FDCS remains limited. For metastatic FDCS, CHOP regimen (cyclophosphamide, doxorubicin, vincristine, and prednisone) and ifosfamide, carboplatin, and etoposide are the most common regimens, although no consensus has been reached [7]. Regarding immune checkpoint inhibitors including pembrolizumab, only one case report has suggested its potential effectiveness for FDCS of the palatine tonsil [18]. Therefore, the benefit of these therapies has not been established, highlighting the critical role of initial treatment.

Recurrence in patients with FDCS of the head and neck is not rare [5, 7]. A retrospective review revealed that the 5-year overall and disease-free survival rates of 76 patients with head and neck FDCS were 81% and 34%, respectively [7]. Moreover, the incidence rates of locoregional recurrence and distant metastasis were 38% and 7%, respectively [7]. In addition, histological features associated with a worse prognosis include size (≥ 6 cm), necrosis, high mitotic count (≥ 5 mitoses per 10 high-power fields), and significant cytological atypia [6, 15]. A high Ki-67 index is associated with poor prognosis [19]. In our case, high rates of Ki-67, a high mitotic count, and significant cytological atypia were observed. Our patient had not experienced recurrence and metastasis 4 years after surgery; however, careful observation over a long period is considered necessary.

In conclusion, we encountered the first case of FDCS of the right palatine tonsil that developed as RIS. Notably, FDCS can develop as RIS over a long-term course after irradiation. Because the diagnosis of FDCS that occurred at the extranodal sites can be challenging, immunohistochemical evaluation of adequate tissue samples is warranted to confirm the diagnosis. When FDCS develops as RIS, an inability to administer PORT may pose a significant problem. Extended resection with a negative margin may be crucial for tumor control, particularly when PORT cannot be administered.

## Data Availability

The authors have nothing to report.
